# Design characteristics, primary stability and risk of fracture of orthodontic mini-implants: Pilot scan electron microscope and mechanical studies

**DOI:** 10.4317/medoral.18674

**Published:** 2013-05-31

**Authors:** André Walter, Heinz Winsauer, Jordi Marcé-Nogué, Sergi Mojal, Andreu Puigdollers

**Affiliations:** 1 Associate professor, Universitat Internacional de Catalunya. School of Dentistry, Department of Orthodontics, PhD candidate, Barcelona, Spain; 2 Private practice, Bregenz, Austria; 3 Researcher and lecturer. Departament de Resistència de Materials i Estructures a l’Enginyeria, Universitat Politècnica de Catalunya, BarcelonaTech, Terrasa, Spain; 4 Instituto municipal de Investigaciones Médicas (IMIM), Statistical analysis and research, Barcelona, Spain; 5 Chairman and professor, Departament of Orthodontics, School of Dentistry, Universitat Internacional de Catalunya, Barcelona, Spain

## Abstract

Objectives: Orthodontic mini-implants (OMIs) are increasingly used in orthodontics but can fail for various reasons. This study investigates the effects of OMI design characteristics on the mechanical properties in artificial bone. Material and Methods: Twelve self-drilling OMIs (2 small, 6 medium, 4 large) from 8 manufacturers were tested for their primary stability in simulated medium-high cancellous bone and the risk to fracture in high-density methacrylate blocks. For the assessments of the maximum insertion torque (IT) and torsional fracture (TF) 5 of each OMI were used and for the pull-out strength (POS) 10. The OMIs were inserted with a torque screwdriver (12 sec/360°) until the bottom at 8 mm depth was reached. OMI designs were analyzed with a scan electron microscope (SEM). Results: SEM images revealed a great variation in product refinement. In the whole sample, a cylindrical OMI shape was associated with higher POS (p<0.001) but lower IT (p=0.002) values. The outer and inner OMI diameters were design characteristics well correlated with POS, IT and TF values (ranging from 0.601 to 0.961). Greater thread depth was related to greater POS values (r= 0.628), although OMIs with similar POS values may have different IT values. Thread depth and pitch had some impact on POS. TF depended mainly on the OMI inner (r= 0.961) and outer diameters (r=0.892). A thread depth to outer diameter ratio close to 40% increased TF risk. Conclusions: Although at the same insertion depth the OMI outer and inner diameters are the most important factors for primary stability, other OMI design characteristics (cylindrical vs. conical, thread design) may significantly affect primary stability and torsional fracture. This needs to be considered when selecting the appropriate OMI for the desired orthodontic procedures.

** Key words:**Orthodontic mini-implants, primary stability, insertion torque, pullout strength, torsional fracture.

## Introduction

The primary implant stability of orthodontic mini-implants (OMIs) is not only affected by the bone quality (ratio compact to trabecular bone) (1), and bone quantity (mineral density) (2), but also by the OMI design characteristics. The latter include length, diameter, thread depth, width, helix angle and pitch (axial distance between threads) variables, thread depth-to-outer diameter ratio, presence of flutes (recessed areas in the cross-sectional area of the OMI that carry bone chips away from the cutting edge as the screw rotates) and body shape (conical, cylindrical). Length and diameter are most important for the OMI primary stability (3,4). Other OMI characteristics are in general not declared by the manufacturers.

The aim of the study was to assess the design characteristics of 12 commercially available self-drilling OMIs using a scanning electron microscope (SEM). Established test procedures were used to investigate the mechanical properties of the OMIs in terms of pullout strength (POS), insertion torques (IT) and, torsional fracture (TF).

## Material and Methods

**Figure 1:**
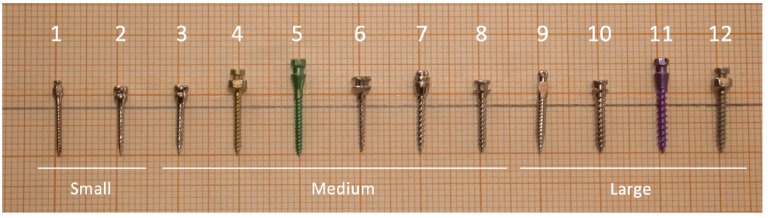
The 12 different OMIs that were investigated in this study.

240 self-drilling titanium (Ti)-alloy (Ti-6AI-4Va) OMIs (12 types from 8 manufacturers) were investigated ( Table 1, Fig. 1). Prior to the mechanical tests, pitch (axial distance between threads), thread (major diameter D2), shank (minor diameter D1) and thread angle were measured by means of SEM imaging (Quantas 200, EEUU). Shank analysis was measured at 25-30x magnification, and thread details at 120x to 140x magnification.

**Table 1:**
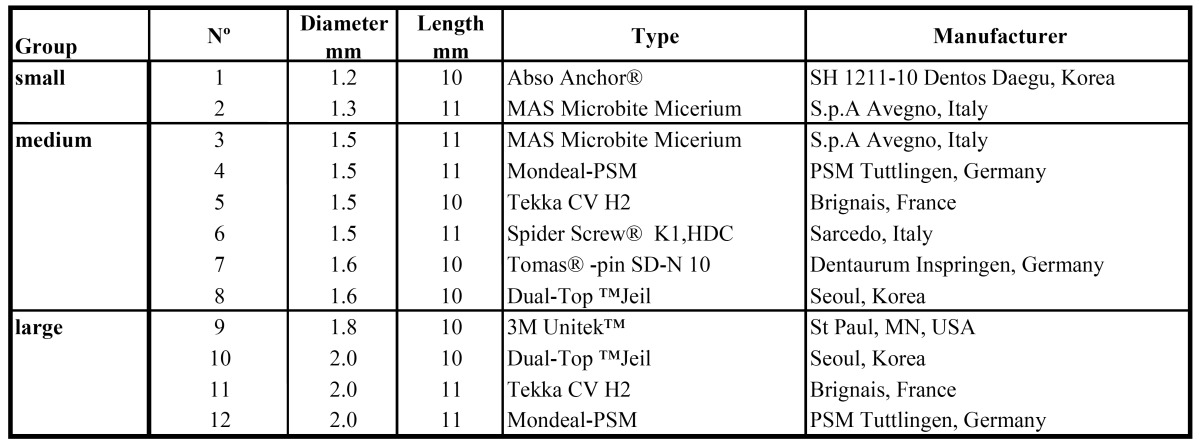
Diameter groups, diameters, lengths, type and manufacturer of the investigated OMIs.

The mechanical tests (POS, IT) were performed as described by others (5-7) using artificial bone simulating high cancellous bone (Sawbone™ Pacific Reasearch Laboratories Inc., EEUU), 120 x 30 x 30mm with a density of 25 pounds per cubic foot; g/cc= 0.40, compression (strength 12.9 Mpa, Modulus 317 Mpa), tension (strength 8.8 Mpa, Modulus 39.9 Mpa) and shear (strength 5.9 Mpa, Modulus 6.8 Mpa) (8). POS and IT were measured by inserting OMIs at 90º into the artificial bone (9) without pre-drilling to a depth of 8 mm (accounting for gingival and bony anchorage support of 3 mm and 5 mm, respectively) at a speed of 12 sec/360. POS tests (10 OMIs of each brand per block) were performed with net axial forces using a Galdabini 1890 servo-hydraulic universal testing machine, Italy. Screw heads were clamped with a custom-made grip. A crosshead speed of 1.0 mm/min. was applied to extract the screws.

In order to measure torque (the clockwise torque resistance IT), screws were inserted perpendicularly (5 OMIs of each brand per block). Torque (Ncm) was measured with the digital torque tester Mecmesin AFT (0 to 500 Ncm, England). Methacrylate blocks (120x90x25) with greater IT resistance were used for the TF tests in order to simulate a high-dense cortical bone thickness (7). TF measurements (5 OMIs per block) were assessed after pre-drilling to a depth of 8 mm (drill diameter 0.5 -1 mm less than the individual OMI’s diameters). Screws were inserted into the pilot hole and tightened (3 sec./ 900) until they fractured.

One-Way ANOVA with Dunnett’s T3 multiple comparison correction were used to detect differences in POS, IT and TF between OMI brands. Spearman’s rho correlation coefficient were calculated to assess relationship between POS, IT, TF and the numerical OMI characteristics. Unpaired Student’s T test was used to compare POS, IT and TF between shank type (cylindrical vs. conical). P values <0.05 were considered as statistically significant. All analysis were performed with SPSS 15.0 (SPSS Inc, Chicago, IL).

## Results

**Figure 2:**
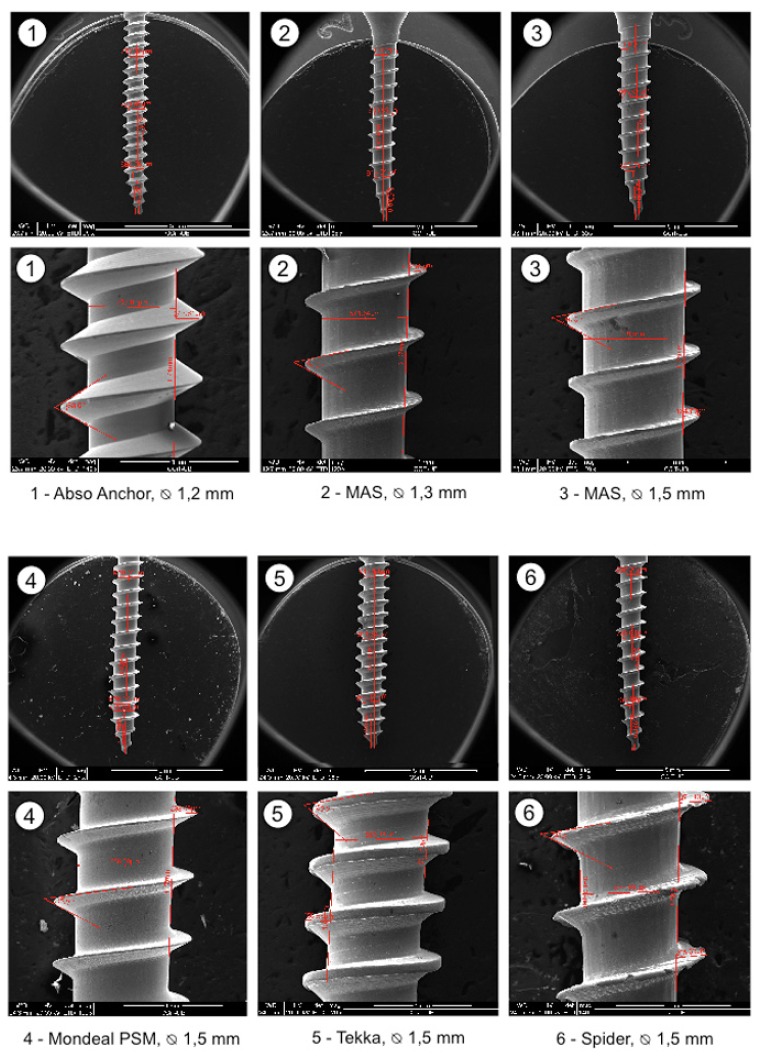
SEM magnifications of the shanks (25-30x) and threads (120-140x) of the 12 different OMIs.

OMIs were grouped into small, median and large according to their diameters. Their design characteristics are summarized in  Table 2. SEM magnifications of the threads show a great variation in product refinements, especially in the medium OMI group (Fig. 2).

**Table 2:**
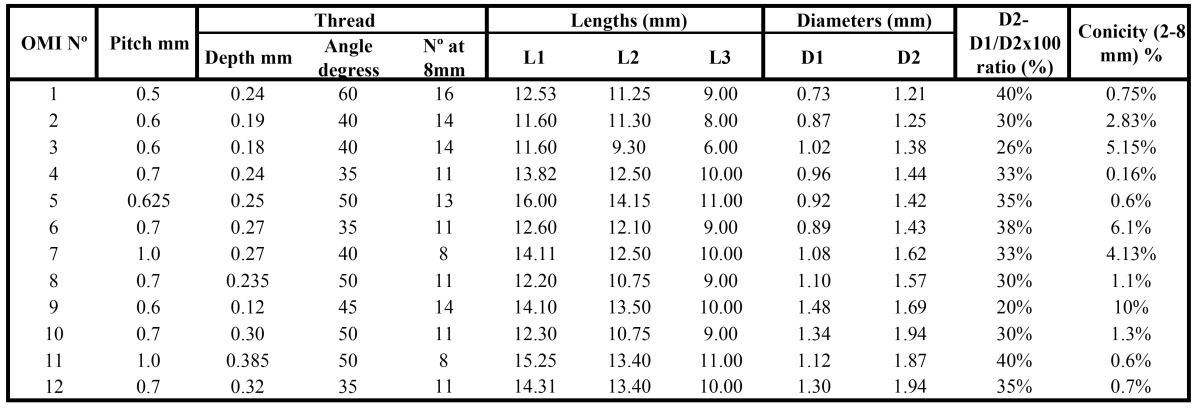
Characteristics and SEM measurements regarding design, length, shape and thread of the 12 different OMIs tested.

**Figure 3:**
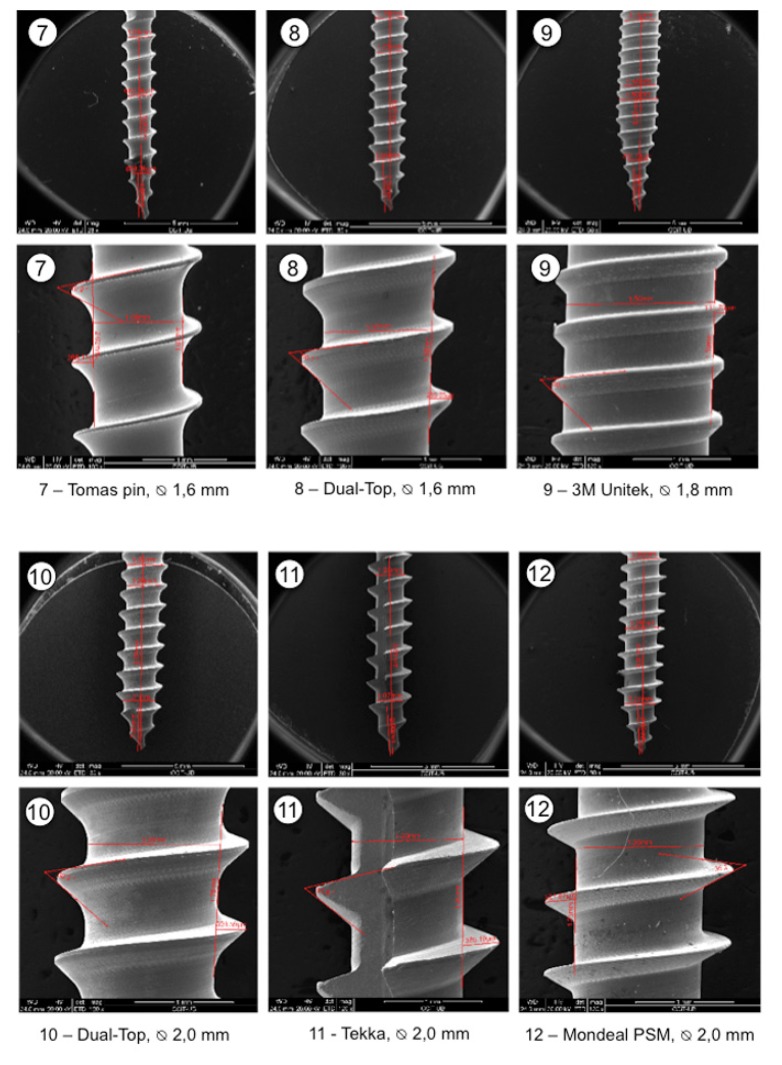
Mechanical test results (mean ± SD) of the pull-out strength, insertion torque and torsional fracture of the 12 different OMIs.

In general, POS values depended on the OMI diameter. However, POS of the smallest OMI (No.1) was close to that of the median group. IT values were either below or around 10 Ncm (Nos. 1,2,4,5,and 6) or higher than 10 Ncm (Nos. 3,7,8,9,10,11, and 12). OMIs Nos. 1, 2 and 6 fractured by a force between 8 to 16 Ncm, while the others fractured by a force between 22 to 48 Ncm (Fig. 3).

In the whole sample, POS values depended on the OMI outer diameter (D2, r=0.846) and IT with the shank (D1, r=0.811) ( Table 3). POS values were higher in cylindrical OMIs (p<0.001), whereas IT values were higher in the conical ones (p<0.002). TF values correlated highly with the screw shank (D1, r=0.961) and the outer diameter (D2, r= 0.892). Statistical signification between pitch and thread depth with POS was high (p<0.001) but the intensity of the correlation of pitch and thread depth with POS was in fact low (r= 0.54 and r= 0.628, respectively). POS was best correlated in the whole sample with the major diameter (D2) and IT with the shank (D1). TF showed levels of correlation with both D1 and D2. The diameters (D1 and D2) showed moderate to high correlations with all mechanical test results.

**Table 3:**
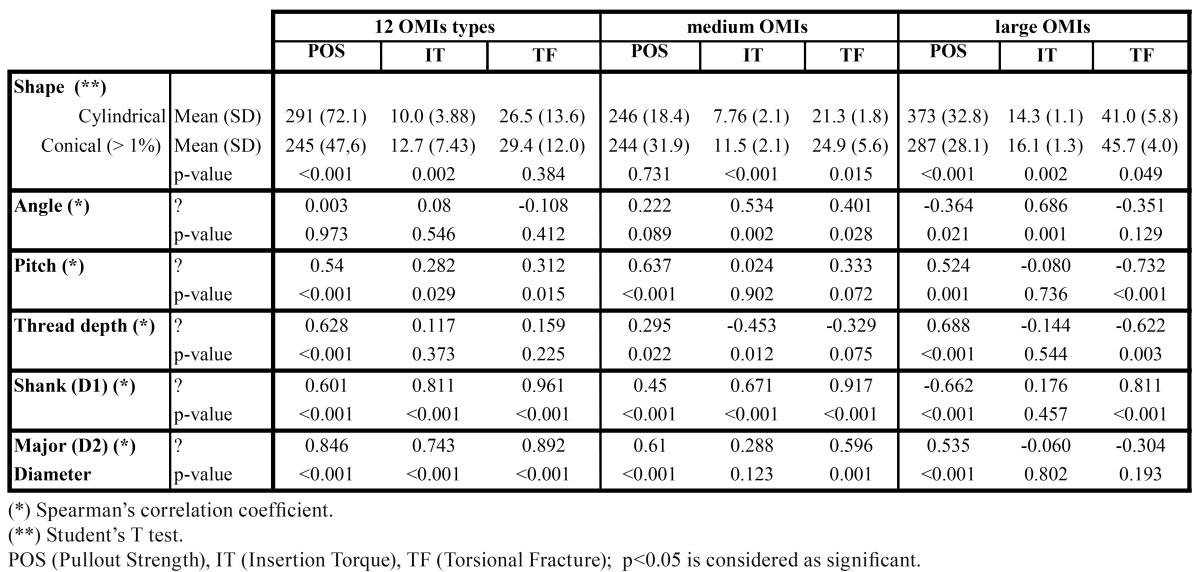
Correlations of total, medium and large OMI design characteristic and mechanical tests.

Within the medium diameter OMI group, conical screws had higher IT (p< 0.001) and TF (p=0.015) values than cylindrical OMIs, although no statistical significant differences in POS has been seen. The greater the angle, the greater was IT (p=0.002) and the greater the pitch, the greater was POS (p<0.001), but correlations were moderate (ρ=0.534 and ρ =0.637, respectively).

Within the large diameter OMI group, conical screws had higher IT values and lower POS values than cylindrical OMIs. In this small group also a trend towards higher TF values was noted for the conical OMIs, but in the limit of the statistical signification. (p=0.049). However, the results need to confirmed in a greater sample. The greater the angle, the greater was IT (p<0.001), but the correlation was only moderate (ρ =0.686). The greater the pitch and the thread depth, the higher was TF (negative correlation) and POS but correlations were moderate to poor being ρ =-0.732 and ρ = -0.622 for TF and ρ =0.524 and ρ =0.688 for POS, respectively ( Table 3). This indicates that OMIs with lower pitch and thread depth are less easy to fracture. Pearson correlations between the results of the different tests of the medium OMI group did not reveal any relation between POS and IT (r =0.054 ,p= 0.774).

## Discussion

Our SEM results show that the OMI production goes along with different refinements. The importance of less accurate production is not yet known but it may well be that metallic detachments may enter the bone (Fig. 2, No 6). The mechanical test results confirm that the overall OMI design has an impact on the mechanical properties. Manufacturers should therefore provide more information on OMIs than solely their lengths and outer diameters so that orthodontists can select the appropriate OMI for the desired procedure. There is no doubt that an increase in outer diameter and length will increase the primary stability of OMIs (Fig. 3) (4). Accordingly, the fracture resistance index increased for each 0.1mm added in outer diameter (10). Our data are compatible with this finding by showing a tendency towards increased fracture risk in small, medium and large OMIs when the thread depth to outer diameter ratio reaches about 40% (reflecting a shank loss of 0.1 or 0.3 mm) (Fig. 3, Nos. 1,6,11). In agreement with the pertinent literature, conical OMIs had higher IT values (11) and slightly lower POS values (5) than cylindrical OMIs. The higher IT values of the taper shape are in general associated with higher compression forces during placement (12) which may result in necrosis of osteocytes and bone resorption. In case of thick cortical bone, pre-drilling may reduce microdamage without affecting the OMI stability (13). By contrast, the use of a narrow drill produced significant bone damage (14). Twelve weeks after OMI placement, the initial difference in primary stability between cylindrical and conical OMIs was no longer seen (15). This indicates that only at the beginning of the orthodontic treatment the OMI shape design is of importance.

Under- or oversized thread pitches being less appropriate for clinical use (1). However, a double thread with decreasing pitch in the neck may counteract this disadvantage by increasing stability due to enhanced interlock surface between OMI and bone (16). An increase in thread angle increased IT. A decrease in thread depth decreased the risk to fracture in the large OMIs group (p=0.003, r =-0.622), with low-moderate correlation (17). This phenomenon is only seen in the large group where differences among pitch and thread depth between specimens are greater than in the median group, a more homogeneous group ( Table 2, Table 3). However, deeper thread depths may increase stabilization in poorer bone quality situations. Large diameter OMIs had higher IT and POS values than smaller OMIs (Fig. 3).

-Small Diameter OMIs: Small diameter OMIs are suitable for narrow interradicular placement as required for simple tooth movements in the maxilla or mandible. OMIs with a small pitch are easier to insert. OMIs with less than 8 mm in length and 1.2 mm in diameter should be avoided due to a high failure rate (18). A high thread depth to outer diameter ratio providing a thinner shank is more prone to TF (Fig. 3, No 1) and less appropriate for clinical use. Immediate loading of small OMIs up to 20 N is possible (18).

Our results confirm that the cylindrical OMI No1 had lower IT values than the conical OMI No 2 (11). Small self-drilling OMIs are not suitable to be inserted into bone with a density greater than 40 pounds per cubic foot (19). The TF value was about half for the cylindrical than the conical (Fig. 3). The force that might fracture the OMI was close to the placement torque indicating that the OMI might already fracture during insertion. The greater thread depth of OMI No1 (ratio 40%) ( Table 2) correspond to the higher POS (Fig. 3) which is favourable in principle, but may not outweigh the increased fracture risk disadvantages. OMI No 2 could therefore be preferable to OMI No 1 for simple tooth movements due to its better characteristics against torsional fracture during insertion.

-Medium Diameter OMIs: Medium diameter OMIs are needed for en masse teeth movement or intrusion of teeth. The greater the length and diameter of the medium OMI, the higher was the implant stability with increasing forces (9). No statistical differences was observed in POS between conical and cylindrical OMIs, but the conical shape were superior in primary stability in terms of IT ( Table 3). OMIs with low thread angles and sharp threads (Fig. 3, No 4) are probably more appropriate for denser bone, e.g. the mandible or the palate due to a lower IT and risk of fracture (Fig. 3). Our results suggest that OMIs the thread depth to outer diameter ratio should not exceed 33% (1 to 3) in order to lower TF risk when OMIs are placed in dense bone. Lower IT values were associated with a trend towards less inflammation in the local bone tissue around the OMIs (20).

Motoyoshi and coworkers (21,22) evaluated a range of 5-10 Ncm as optimum IT for pre-drilling OMIs in both, the maxilla and mandible. For self-drilling OMIs, the adequate IT may be somewhat higher (20). Using conical OMIs, especially Nos. 3,7 and 8, a pilot drill is recommended to decrease the IT for better secondary stability (3) because high placement torques although they increase primary stability may are not favorable in the clinical setting (23). Bone remodelling was shown to counteract the OMI primary stability already 3 weeks after the OMI placement (15). In accordance to this, the removal torque exceeded the placement torque at the end of 10 months of orthodontic treatment (24).

The resistance to extraction of OMIs depends on various factors including OMI design, shear strength (secondary stability) and bone density. Migliorati and coworkers (25) have demonstrated that the thread depth and the pitch are also predictors for the load values in pullout test. We suggest that the ideal thread depth to outer diameter ratio should be around 30% or less to avoid weakness of the shank (Nos 3,8). A sharp low thread angle is desired to reduce placement torque and risk of fracture (No 4). In case of dense bone, a cylindrical shape OMI or a less conical shape OMI with sharp threads is preferable to avoid excessive friction and compression. The conical Dual Top screw (No 8) although mechanically superior to other OMI types with respect to its high IT and stiffness had the greatest resistance to fracture in this group (Fig. 3) (26). Cylindrical OMIs showed no differences in POS values but significantly lower IT values compared to conical OMIs ( Table 3).

-Large Diameter OMIs: Our data confirm that large diameter OMIs with conical design have higher IT values but provide lesser primary stability in terms of POS than large OMIs with a cylindrical design (11) ( Table 3). An increases insertion torques are directly related to cortical bone thickness (27). However, very high placement torques will increase tensile and compressive stress to the peri-implant bone tissue, thus, being of disadvantage. The cylindrical OMI shape superiority was evident in the holding power. In general terms, the TF variable of the 2.0 mm OMIs is 60% higher compared to the median group.

OMIs with a diameter of 2 mm or more showed a 1.8-fold lower risk of failing compared to OMIs with 1.2 mm diameter (28). Immediate or early loading up to 2 N had no impact on OMI stability (18).

The domaine of large and long diameter OMIs is the anchorage for molar distalizers or maxillary bone-born hybrid hyrax expanders (29) requiring higher loading forces. Long flutes along the axis and deep thread depth around 40% of such screws (No. 11) may increase the fracture risk (37.08 Ncm, the lowest of its group; Fig. 3). Short bowl flutes that store squeezed chips may, however, be of advantage due to a decrease in placement torque and increased bending strength.

In our study OMI No.10 fractured during insertion at a mean of about 44 N (depth 8 mm). This is close to the observation by Mischkowski and coworkers (26). They observed head fractures of this OMI at 53-56 N at a depth of 10 mm. It remains to be assessed why in the study by Wilmes and coworkers (17) these screws fractured within the force range of 23 -41 N (total number of screws tested not stated).
